# Cellular Organelles Involved in Hepatitis E Virus Infection

**DOI:** 10.3390/pathogens10091206

**Published:** 2021-09-17

**Authors:** Xing Liu, Menghang Wang, Xin Yin

**Affiliations:** State Key Laboratory of Veterinary Biotechnology, Harbin Veterinary Research Institute, Chinese Academy of Agricultural Sciences, Harbin 150069, China; liuxingshenglin@163.com (X.L.); wmhtcs@163.com (M.W.)

**Keywords:** hepatitis E virus, cellular organelles, life cycle

## Abstract

Hepatitis E virus (HEV), a major cause of acute hepatitis worldwide, infects approximately 20 million individuals annually. HEV can infect a wide range of mammalian and avian species, and cause frequent zoonotic spillover, increasingly raising public health concerns. To establish a successful infection, HEV needs to usurp host machineries to accomplish its life cycle from initial attachment to egress. However, relatively little is known about the HEV life cycle, especially the functional role(s) of cellular organelles and their associated proteins at different stages of HEV infection. Here, we summarize current knowledge regarding the relation of HEV with the different cell organelles during HEV infection. Furthermore, we discuss the underlying mechanisms by which HEV infection is precisely regulated in infected cells and the modification of host cell organelles and their associated proteins upon HEV infection.

## 1. Introduction

Hepatitis E Virus (HEV), a single-stranded, positive-sense RNA virus, is now recognized as a significant cause of acute viral hepatitis in both developing and industrialized regions, leading to 20 million infections, more than 3 million cases of hepatitis E, and 70,000 deaths annually [1,2]. It is usually transmitted via the fecal–oral route either by consumption of contaminated food or water or by direct person-to-person contact [3,4,5]. The HEV within the Orthohepevirus A genus has been subdivided into at least 8 genotypes. Genotypes 1 and 2 have been detected exclusively in humans, whereas genotypes 3 and 4 circulate among humans and other animal species including pig, deer, rabbit, monkey, cow, goat, and wild boar [6,7,8,9,10,11,12]. Genotypes 5 and 6 infect wild boar without reports showing zoonotic transmission to humans [13]. Genotypes 7 and 8 are known to infect dromedary and Bactrian camels with potential zoonotic risk [14,15]. In recent years, the reported cases of HEV infection have been steadily increasing globally, especially in developed countries [16]. Despite this public health threat, no specific treatment modalities are available currently [17,18]. Therefore, deepened understanding of the molecular underpinnings of HEV replication cycle, as well as of the molecular interactions with host cellular machineries, is critical to developing novel therapeutic interventions against HEV infection.

**Figure 1:**
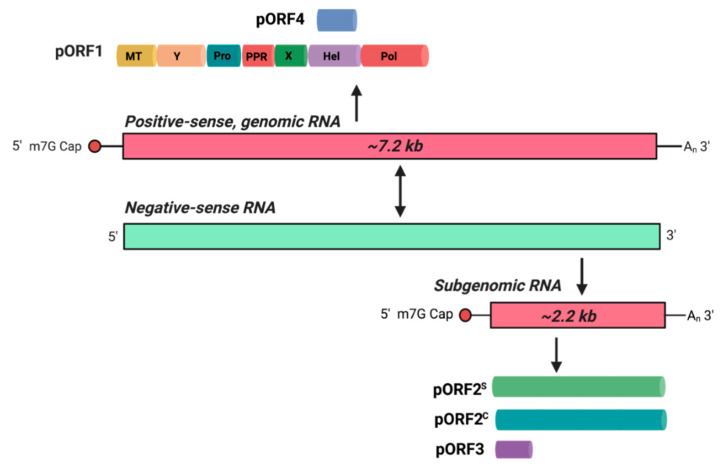
HEV genome, replication, and translation. Hepatitis E virus (HEV) has an ~7.2kb, positive-sense (+) single-stranded genomic RNA, which contains ORF1, ORF2 and ORF3 as described below, plus ORF4, an additional ORF unique to HEV genotype 1 that translates pORF4 promoting virus infection upon endoplasmic reticulum (ER) stress stimulation. Genomic RNA has a 7-methylguanosine (m7G) Cap at the 5’ end and is polyadenylated at the 3’ end. After viral entry and uncoating, the positive-sense full-length viral genome is translated by host ribosomes to produce a polyprotein pORF1 that contains Met, Hel, RdRp, ‘Y’, ‘X’, ‘HVR’, and putative PCP domains. pORF1 transcribes a negative-sense (−) intermediate RNA from the positive-sense strand. The negative-sense strand then serves as a template for the transcription of numerous positive-sense RNA genomes for packaging into new progeny virions, as well as an ~2.2 kb subgenomic RNA (sgRNA) containing ORF2 and ORF3, while ORF3 entirely overlaps with ORF2 except for one leading base pair. The sgRNA is also capped at the 5ʹ end and polyadenylated at the 3ʹ end, and translates into the capsid protein (pORF2^C^), secreted antigen (pORF2^S^), and protein ORF3 (pORF3) via a leaky scanning mechanism.

HEV has long been known to be a non-enveloped virus since its discovery in the 1980s [19]. Recently, a membrane-associated, quasi-enveloped form of virus particles (eHEV) was identified in the bloodstream of infected individuals and culture supernatants, which mediates virus spread within the host [20], while non-enveloped virions are present in feces of infected patients for stable transmission from person-to-person [21,22]. Notably, unlike classical enveloped viruses such as influenza viruses, hepatitis C virus (HCV), and zika virus, which have surface viral proteins embedded in the lipid membrane, eHEV has no viral antigens on its surface and is resistant to neutralizing antibodies [23,24,25]. The presence of two different virus particles in infected individuals demonstrates that HEV possesses a complex dual life cycle to execute distinct functions for the establishment of successful infection. The 7.2 kb genome of HEV contains a 7-methylguanosine RNA cap at the 5′ end and polyadenylation at the 3′ terminus, as well as three conserved open reading frames (ORFs) termed ORF1, ORF2, and ORF3 [26] (Figure 1). ORF1 encodes a nonstructural polyprotein, pORF1, which contains the methyltransferase (Met), RNA helicase (Hel) and RNA-dependent RNA polymerase (RdRp), along with several non-enzymatic regions (the ‘Y’, ‘X’, and ‘hypervariable’ (HVR) regions) and a putative papain-like cysteine protease (PCP) [27]. ORF2 encodes a glycosylated protein function as secreted antigen (pORF2^S^) and a capsid structural protein (pORF2^C^) separately [28]. ORF3 encodes a small multifunctional palmitoylated phosphoprotein required for HEV egress from infected cells [29,30,31]. In addition to these three conserved ORFs, the genotype 1 HEV harbors an ORF4, which encodes a protein via an internal ribosome entry site (IRES)-like element in response to endoplasmic reticulum (ER) stress [32]. 

The initial step of the HEV life cycle begins with the specific binding of the virions to the yet unidentified cellular receptors [33]. Given the morphological differences between non-enveloped virus particles and eHEV, it is believed that they utilize distinct pathways to enter target cells [34]. Following internalization, the incoming viral genomes act as templates to produce non-structural polyproteins pORF1 and negative-stranded RNA intermediates for forming the replication and transcription complex [26]. Concordant with the expression of pORF1 from the 7.2 kb full-length genome [27], the pORF2^C^ and viroporin pORF3 are translated from a bicistronic 2.2 kb subgenomic viral RNA to encapsulate the newly produced genomic RNA for assembly [35,36]. In addition, pORF2^S^ was recently identified to be translated from the same bicistronic subgenomic RNA through a leaky scanning mechanism, while the functional role(s) of the pORF2^S^ is still under investigation [28]. Finally, eHEV are egressed from infected cells in an exosomal pathway-dependent manner [37]. Host cellular organelles are principally thought to determine the complete viral life cycle [38]. Nevertheless, the detailed functions of the cellular organelles and their associated proteins remain largely unknown due to the lack of an efficient cell culture system for HEV infection [39]. In this review, we summarize the findings that shape our current understanding of host determinants involved in HEV life cycle, including binding, entry, trafficking, replication, assembly, and release.

## 2. Cellular Organelles and Proteins Participating in HEV Binding, Entry, and Uncoating

### 2.1. Plasma Membrane and Membrane-Associated Proteins That Mediate HEV Binding and Entry

**Figure 2:**
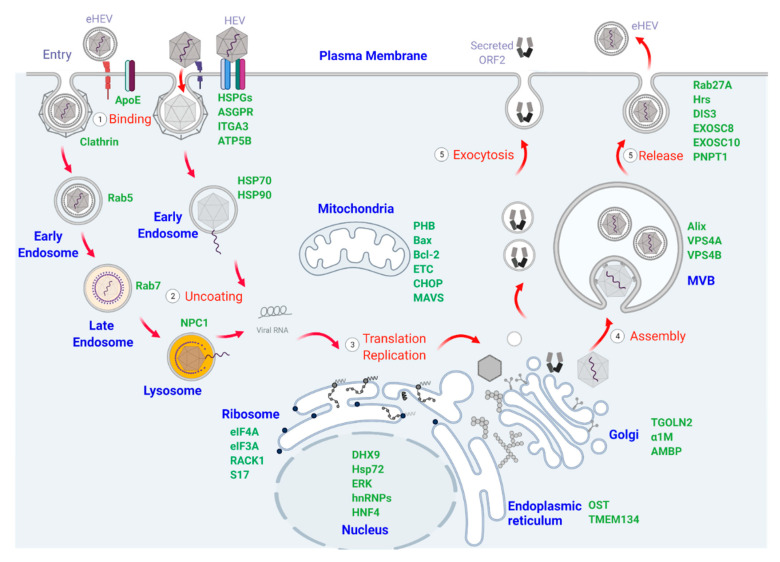
The replicative life cycle of hepatitis E virus. The life cycle of quasi-enveloped HEV (eHEV) and non-enveloped HEV initiates with the binding with cellular membrane proteins, such as ApoE or HSPGs, ASGPR, ITGA3 or ATB5B, respectively to allow virus **entry** into cells. Both eHEV and HEV are believed to rely on clathrin-mediated endocytosis for internalization. eHEV is **trafficked** through early- (Rab5+) and late- (Rab7+) endosomes and eventually to lysosomes harboring NPC1 for the **uncoating** and release of the viral genome into the cytoplasm, while HEV is thought to inject the viral genomes from early endosome to cytoplasm. Subsequently, **translation** of the pORF1 from incoming viral genomes allows **replication** to proceed with **transcription** of the 7.2 kb genomic and the 2.2 kb subgenomic RNA through a negative-strand RNA intermediate (-), and **translation** of the subgenomic RNA to produce the ORF2 and ORF3 encoded proteins in ER (associated with proteins of OST and TMEM134) and ribosome that is promoted by eIF4A, eIF3A and RACK1. Mitochondrial proteins, such as PHB, Bax, Bcl-2, CHOP, MAVS are involved in shaping the microenvironment during HEV replication. HEV infection in cells also triggers the shuttle of host proteins between nucleus and cytoplasm. Secreted ORF2 protein (pORF2^S^) is translated from the same bicistronic subgenomic RNA through leaky scanning mechanism and modified in Golgi; then, it is transported outside by **exocytosis** in dimer. The non-glycosylated ORF2 protein (pORF2^C^) forms naked virions by **self-assembling** to capsid and packaging of the viral genome, while eHEV formation requires the engagement of pORF3 and the coating of lipid membrane in multivesicular bodies. **Release** of eHEV involves exosomal-associating proteins Rab27A, Hrs, DIS3, EXOSC8, EXOSC10, and PNPT1, and fusion with the plasma membrane. Secreted particles remain associated with the lipid membrane in the culture supernatant of infected cells, while HEV remains in cells.

Plasma membranes are complex architectures consisting of hundreds of lipids and proteins to separate the cell interior from the outside environment [40]. Viruses must hijack the molecules on the plasma membrane to cross membrane barriers; thus, specific interactions between virions and cellular receptors are the first event resulting in the injection of the viral genome into the cytoplasm for productive infection [41]. The molecules with proved relevance for non-enveloped HEV binding include heparin sulfate proteoglycans (HSPGs) [42,43], asialoglycoprotein receptor (ASGPR) [44], and integrin α3 (ITGA3) [45], as shown in Figure 2. Nonetheless, none of them fulfill true bona fide criteria of the virus receptor.

HSPGs, an abundant molecule on the cellular surface of most mammalian cells, served as non-specific attachment factor to facilitate the subsequent interaction between virions with specific receptors [46,47]. Two potential sugar binding sites were mapped in P1 and P2 domains at the HEV capsid protein interface [33]. In addition, removal of cell surface heparan sulfate significantly reduced HEV capsid binding, indicating HSPGs are required for non-enveloped HEV entry [42]. eHEV differ structurally from non-enveloped virions. As expected, HSPG was not required for eHEV infection, suggesting that no potential HSPG binding site existed on the surface of eHEV [34]. 

Apoliprotein E (ApoE), a core component of plasma lipoproteins that mainly functions in lipoprotein-mediated lipid transport in plasma was found to be upregulated in HEV-infected swine liver by proteomic analysis [48]. Being essential for the transport of cholesterol into and out of the liver, ApoE may be essential for eHEV entry. As reported previously, ApoE participates in the binding of HCV to specific receptors through its interaction with HCV envelope glycoprotein E2 [49]. More importantly, a study showed that single-nucleotide polymorphisms (SNPs) of ApoE potentially associate with protection against HEV infection in a cohort study [50]. However, another study found that HEV RNA replication and viral production were not affected by ApoE polymorphisms, at least in an Huh-7.5 cell culture model [51].Therefore, the detailed mechanism underlying the observation needs to be further investigated.

ASGPR and ITGA3 that predominately present on the cellular membrane were also identified as dependent factors required for HEV entry and trafficking via different approaches [44,45]. siRNA-mediated depletion could significantly reduce HEV binding on the cellular surface, indicating that both ASGPR and ITGA3 act primarily as attachment factors to facilitate HEV entry [44,45]. In addition, HSP90 [52] and ATP5B [53] were shown to directly bind with HEV capsid to mediate intracellular trafficking of incoming virions, but not the binding of HEV virions on the cellular surface. Therefore, these host factors seem to participate in HEV entry at different stages. However, the functional validation of these dependent factors in the context of authentic virus infection is still lacking.

### 2.2. Endosomal Vesicles and Related Signaling Pathways Involved in HEV Trafficking

Endosomes are membrane-bound endocytic organelles inside cells that play key roles in the sorting and delivery of cargos to various intracellular destinations [54]. Both eHEV and non-enveloped HEV heavily rely on endosomal vesicles to deliver the viral genome into infected cells [34]. Our previous study demonstrated that clathrin-mediated endocytosis (CME), which is a common pathway exploited by many viruses [55,56], served as the main entry route for both virions to enter the host cells [34]. Depletion of the core components of CME resulted in reduced infectivity of both eHEV and non-enveloped HEV in hepatocytes.

In terms of eHEV internalization and trafficking, the small GTPases Rab5 and Rab7 were required [34], suggesting that eHEV moves across the entire endolysosomal network. After endocytic internalization and endosomal sorting, the internalized eHEV was targeted to the lysosome that had the proper environmental conditions to trigger its membrane degradation, resulting in access of the exposed capsid to intracellular receptors for eventual uncoating [34]. Niemann-Pick C1 (NPC1), a lysosomal cholesterol transporter, was verified to be essential for efficient infection of eHEV, as depletion of NPC1 reduced eHEV but not HEV infectivity in hepatocytes [34]. More recently, it was shown that phosphatidylserine receptor-Hepatitis A Virus Cellular Receptor 1 (HAVCR1) and NPC1 participated in quasi-enveloped hepatitis a virus (HAV) infection by an undefined mechanism that mediates the delivery of genetic material into the cytoplasm [57]. Therefore, the HAVCR1-NPC1 pathway appears to represent a common mechanism for cell entry of both quasi-enveloped HAV and eHEV. 

Non-enveloped HEV has been established to rely on CME as the main entry route to enter hepatocytes [34]. Subsequently, non-enveloped HEV stop at an undefined compartment near plasma membrane for uncoating. Further research is required to identify the virus-specific factors responsible for the entry and trafficking of both non-enveloped HEV and eHEV. Given the rapid innovation in live-imaging techniques and labeling of virus components, the detailed functions of membrane-associated proteins in HEV entry will be uncovered shortly.

## 3. Cellular Organelles and Proteins Involved in HEV Translation and Replication

### 3.1. Endoplasmic Reticulum and ER-Resident Proteins Involved in HEV Translation and Replication

The ER is a membranous system that mediates the biosynthesis of membrane and secreted proteins, as well as lipids such as fatty acids, sphingolipids, phospholipids, and cholesterol [58,59]. The synthesis of approximately one-third of all cellular proteins is governed in this organelle [58]. As obligate parasites that solely rely on host machineries to thrive and produce progeny virions, almost all viruses usurp the endogenous functions of numerous ER-resident channels, chaperones, and enzymes throughout the whole viral life cycle [60,61,62,63]. Confocal imaging revealed that the overexpressed non-structural polyprotein pORF1 predominately co-localized with ER marker BAP31 [64], indicating that ER is likely to be a central organelle that governs HEV replication. As a positive-sense RNA virus, after uncoating, pORF1 is immediately translated from the 7.2 kb RNA genome to produce RdRp for viral RNA synthesis [26]. Thus, the ER localization of pORF1 indicated that ER may act as a viral factory for the synthesis of the key viral components.

Multiple ER-associated proteins interacting with HEV encoded proteins were discovered to modulate viral replication upon infection [31,32,64,65,66,67,68,69]. It seems that ER-resident enzymes, such as the oligosaccharyl transferase (OST) complex, drive the attachment of glycan moieties to the nascent pORF2^S^ for glycosylation modification [70,71]. Treatment with Brefedin A, a protein transport inhibitor, dramatically reduced glycosylated ORF2 protein secretion [72]. The folded and glycosylated ORF2 proteins then exit the ER by packaging into coat protein complex II (COPII)-coated vesicles, and then transit through the classical secretory pathway en route to efficient secretion. Intriguingly, the ER-associated degradation pathway was also reported to regulate the retro-translocation of pORF2^S^ from the ER lumen to the cytosol without validation in the context of bona fide infection [70]. Transmembrane protein 134 (TMEM134), an ER-associated protein, was identified as a partner of pORF2^C^ via a split-ubiquitin yeast two-hybrid screening [68]. It is proposed that TMEM134 negatively regulates pORF2^C^-mediated inhibition of the NF-κB signaling pathway [68].

pORF1 and pORF3 contain multiple predicted palmitoylation sites. Removal of the putative palmitoylation sites at residues C336-C337 of pORF1 protein was lethal to HEV infection in HepG2/C3A [73]. The HEV variant with mutations at the palmitoylation sites within pORF3 lost its ability to egress from infected cells [29]. Therefore, palmitoylation modification is essential for HEV infection via maintaining the stability and functions of pORF1 and pORF3 [29,73]. As reported previously, the zinc finger Asp-His-His-Cys (DHHC) domain-containing palmitoyltransferases (ZDHHCs) that display ER and/or Golgi localization govern palmitoylation modification [74]. It remains to be determined which ER-resident ZDHHCs is responsible for processing.

ER stress stimulated by thapsigargin or tunicamycin induces a cap-independent, internal initiation-mediated translation of a novel viral protein known as pORF4 in HEV-infected cells [32]. The pORF4 protein is specifically encoded by genotype-1 HEV and directly interacts with eEF1α1 to stimulate RdRp activity, leading to enhanced replication [32]. These results indicate that the ER and ER-resident proteins play essential roles in the HEV life cycle via modulation of virally encoded proteins.

### 3.2. Ribosomes and Associated Factors Necessary for Translation of HEV Proteins

Ribosomes are central apparatuses that catalyze protein synthesis [75]. The synthesis of viral proteins heavily depends on the functions of the host ribosomes [76]. A set of host translation factors such as eIF4A, eIF3A, and ribosomal protein receptor for activated C kinase 1(RACK1) were identified as key players supporting HEV replication [77]. Translation initiation protein eIF4A forms the eIF4F translation initiation complex together with the large scaffolding protein eIF4G and the cap-binding protein eIF4E to drive the cap-dependent translation initiation [78]. The natural compound silvestrol, a specific inhibitor of eIF4A, exhibited a potent antiviral effect against HEV replication in vitro and in vivo via preventing enzymatic unwinding of eIF4A [79,80]. RACK1, a protein of the 40S ribosomal subunit, promoted translation of HCV and poliovirus [81,82]. Upon protein kinase C-mediated stimulation, activated RACK1 initiated the following: PKCβII complex phosphorylates eIF4G at S1093 in the tight 48S initiation complex, possibly facilitating dissociation/recycling of eIF4F [83]. However, the precise role of RACK1 in HEV replication remains unclear. 

In addition to regulating viral protein translation, the insertion of a ribosome protein sequence appears to play vital roles in mediating cross-species infection by certain HEV strains. Insertion of a 171-nucleotide sequence encoding amino acids 21 to 76 of the human ribosomal protein S17 within the hypervariable region (HVR) of HEV pORF1 contributed to the adaptation of HEV strain Kernow C-1 P6 in cell lines from different animal species [84]. Furthermore, lysine residues within the human ribosomal protein S17 sequence were responsible for enhanced virus replication [85]. Notably, an RNA sequence encoding ribosomal protein S19 was also found in the HVR of the HEV GT3 strain LBPR-0379 [86]. These observations suggest that HEV viral quasi-species capable of enhanced levels of virus replication can be produced by the insertion of RNA sequences encoding cellular ribosomal proteins. The mechanisms underlying the enhanced levels of HEV replication are still unknown.

### 3.3. Mitochondria and Related Signaling Participating in HEV Infection

Mitochondria is a double membrane intracellular organelle that plays multiple important roles in maintaining homeostasis [87]. Increasing evidence demonstrates that mitochondria plays vital roles in antiviral immune responses [88], apoptosis [89], and inflammation [90] induced by virus infection. HEV infection in Mongolian gerbils caused mitochondria swelling and vacuolation via ultrastructural pathological analysis [91]. The mitochondrial damage triggered the apoptosis signaling pathway, leading to the necrosis and cell death of renal epithelial cells in the acute phase of HEV infection [91]. In addition, loss in mitochondrial cristae and swollen mitochondria were observed in HEV-infected hepatocytes via transmission electron microscopy [92]. These results suggest that mitochondrial lesions may be biomarkers of HEV infection. 

Quantitative proteomics analysis found that prohibitin (PHB), a critical mitophagy receptor mediating autophagic degradation of mitochondria, was upregulated in HEV-infected livers in a swine model [48]. Pro-apoptotic protein BCL2-associated X protein (Bax) and B-cell lymphoma 2 (Bcl-2), mitochondrion-mediated apoptosis regulating proteins, were also induced in the HEV infected gerbils, resulting in the activation of mitochondrial apoptotic pathway and apoptosis [93]. Electron transport chain (ETC), a key component of the mitochondria, positively regulates HEV replication [94]. Pharmacological inhibition of complex III of ETC restricted the replication of HEV [94]. Thus, ETC could be a viable anti-HEV target for therapeutic development. The pro-apoptotic gene C/EBP homologous protein (CHOP) was reported to be activated by pORF2^C^ [65]. CHOP mediates translocation of Bax from cytosol to mitochondria [95]. However, direct evidence is still missing regarding the function of CHOP in HEV replication.

Adaptor protein MAVS, which is crucial for initiating the activation of antiviral innate immune response to RNA virus infection, negatively regulated HEV infection [96]. Unlike other hepatotropic viruses, including HAV and HCV, which are capable of cleaving MAVS to suppress the signaling activation [97,98], HEV does not target MAVS for degradation [99], but instead disrupts JAK-STAT1 signaling to block interferon-induced genes’ (ISGs) expression [100,101]. Therefore, mitochondrial dynamics modified by HEV infection determines the outcome of infection.

### 3.4. Interactions between HEV Components and the Nucleus during Virus Infection

HEV appears to complete its whole life cycle outside the nucleus [102]. Surprisingly, HEV pORF2^C^ was detected in the nucleus by immunohistochemistry; thus, HEV pORF2^C^ probably associates with nuclear components to regulate viral infection [103]. In addition, DExH-box helicase 9 (DHX9), which localizes in both the nucleus and the cytoplasm, interacts with HEV 3’UTR to function as a transcriptional regulator [104]. 

HEV infection seems to trigger the shuttle of the host proteins between nucleus and cytoplasm [65,105,106,107,108]. HEV pORF2^C^ reportedly interacted with Hsp72 and mediated its nuclear accumulation [65]. The extracellular signal-regulated kinase (ERK), a member of the MAP kinase family of enzymes, displayed enhanced activity and nuclear localization mediated by pORF3 [105]. pORF3 also impaired nuclear translocation of hepatocyte nuclear factor 4 (HNF4) by increasing its phosphorylation through the ERK and Akt kinases, causing down-regulation of HNF4-responsive genes in pORF3-expressing cells [108]. Heterogeneous nuclear ribonucleoproteins (hnRNPs), namely hnRNPK, hnRNPA2B1, hnRNPH, PCBP1, and PCBP2, redistributed from nucleus to cytoplasm in HEV-infected cells [106]. hnRNPK and hnRNPA2B1 interacted with the promoter regions of HEV RNA and HEV polymerase protein to increase HEV RNA replication, while hnRNPH, PCBP1, and PCBP2 only bound with HEV genomic promoter to inhibit viral replication [106]. Consistently, quantitative proteomics analysis showed that hnRNPK was upregulated in HEV-infected cells, indicating that HEV requires plenty of hnRNPK for its efficient replication. However, the outcome of protein shuttling between cytosol and nucleus in HEV life cycle is still unclear. Novel proximity labeling techniques can be applied to dissect the temporal and spatial localization of host proteins in response to HEV infection.

## 4. Cellular Organelles and Proteins Involved in HEV Assembly and Release

### 4.1. The Role of the Golgi Apparatus in HEV Assembly

The assembly process of HEV viral particles has garnered much attention, but it is still largely unknown [33,109,110,111,112,113,114]. The pORF2^C^ undergoes post-translational modification in Golgi and self-assembles to capsid, and binds with HEV full-length genomic RNA for encapsulation [115]. The arginine-rich domain in the N-terminal region of the capsid protein and the 5’ end of the viral genomic RNA were demonstrated to be responsible for the assembly [116,117]. However, host proteins involved in HEV assembly have yet not been identified. pORF2^S^ serves as a viral secreted antigen with unidentified biological function; it is translated from the same bicistronic subgenomic RNA through a leaky scanning mechanism and post-translated in Golgi, and is then transported outside by exocytosis in dimer [28].

The Golgi apparatus plays a central role in protein transport by regulating cargo sorting and trafficking [118]. Trans-Golgi network protein 2 (TGOLN2), an intracellular protein derived from the trans-Golgi network, was enriched on the lipid membrane of eHEV particles [119]; thus, the Golgi apparatus probably participates in the assembly of eHEV. Importantly, the formation of the eHEV recruits pORF3, which is post-translated by phosphorylation and palmitoylation in Golgi. The modified pORF3 may mediate the incorporation of lipid membrane in multivesicular bodies (MVBs) [31,35,36]. Liver-specific α1-Microglobulin (α1m) was found to re-distribute in the Golgi compartment in HEV ORF3-expressed cells. The HEV pORF3 interacted with α1m and its precursor α1m/bikunin precursor (AMBP), mediating the transport [120]. However, the roles of these host proteins in HEV assembly remain unclear. Future study is required to solve questions regarding HEV assembly.

### 4.2. Multivesicular Bodies (MVB) and Exosomal Pathways in HEV Egress

The mechanism underlying HEV egresses from infected cells remains to be determined. Previous studies demonstrated that MVB sorting and the exosomal pathway are key players mediating HEV release from infected cells [30,121,122]. Tumor susceptibility gene 101 (Tsg101), a component in the endosomal sorting complex required for transport (ESCRT) machinery, interacted with HEV pORF3 via the PSAP late domain [123]. In addition, components in the ESCRT complex, such as apoptosis-linked gene 2-interacting protein X (ALIX), VPS4A, and VPS4B, were involved in HEV egress from infected cells [121]. Depletion of either ALIX, VPS4A, or VPS4B decreased the budding efficiency of HEV [121].

eHEV resemble exosomes in size range 50–100 nm from infected cells [37]. GW4869, an inhibitor of exosome biogenesis, blocked HEV egress from HEV-infected cells, indicating that HEV hijacks and customizes the exosomal pathway to promote its budding. Depletion of Rab27A or Hrs, the regulators of exosome secretion, led to reduced HEV budding from infected cells [122]. Rat HEV egress was also suppressed in Rab27A- or Hrs-depleted cells [121]. Notably, the expression levels of the key components of the exosomal pathway, such as exosome endoribonuclease and 3’-5’ exoribonuclease (DIS3), exosome component 8 (EXOSC8), exosome component 10 (EXOSC10), and polyribonucleotide nucleotidyltransferase 1 (PNPT1), were elevated in response to HEV infection [104]. Although there is no compelling evidence that supports such a notion, cell lysis may advance the release of non-enveloped HEV as an additional mechanism. Therefore, the majority of HEV particles egress from infected cells by hijacking the exosomal pathway. 

## 5. Conclusions and Perspectives

In the last decade, several aspects regarding the life cycle have been propelled forwards by the development of a cell culture system and small animal models. A better understanding of the roles of host organelles and their associated proteins in the HEV life cycle is critical for understanding the pathogenesis and guiding novel strategies for therapy. Therefore, future studies that aim to delineate the cellular receptors, the interaction network between HEV encoded protein, and host proteins are warranted. Although ribavirin therapy is favorable clinically, more active compounds are still urgently needed. Drugs that can directly disrupt the functions of host machinery required for HEV infection should be considered.
